# Unexpected partial correction of metabolic and behavioral phenotypes of Alzheimer’s *APP/PSEN1* mice by gene targeting of diabetes/Alzheimer’s-related *Sorcs1*

**DOI:** 10.1186/s40478-016-0282-y

**Published:** 2016-02-25

**Authors:** Elysse M. Knight, Henry H. Ruiz, Soong Ho Kim, Jessica C. Harte, Wilson Hsieh, Charles Glabe, William L. Klein, Alan D. Attie, Christoph Buettner, Michelle E. Ehrlich, Sam Gandy

**Affiliations:** Departments of Neurology and Psychiatry, and Alzheimer’s Disease Research Center, Icahn School of Medicine at Mount Sinai, New York, 10029 USA; Diabetes, Obesity, and Metabolism Institute, Department of Medicine, Icahn School of Medicine at Mount Sinai, New York, NY 10029 USA; Universty of California, Irvine, CA 92697 USA; Department of Neurobiology, Northwestern University, Evanston, IL 60208 USA; Department of Biochemistry, University of Wisconsin, Madison, WI 53706 USA; Departments of Neurology and Pediatrics, Icahn School of Medicine at Mount Sinai, New York, 10029 USA; James J. Peters Veterans Affairs Medical Center, Bronx, New York, 10468 USA; Biochemistry Department, Faculty of Science and Experimental Biochemistry Unit, King Fahd Medical Research Center, King Abdulaziz University, Jeddah, Saudi Arabia

**Keywords:** Alzheimer’s disease, Amyloid beta, Metabolic, Insulin, Glucose, Transgenic model

## Abstract

**Introduction:**

Insulin resistance and type 2 diabetes mellitus (T2D) are associated with increased risk for cognitive impairment, Alzheimer’s disease (AD) and vascular dementia. *SORCS1* encodes a protein-sorting molecule genetically linked to both T2D and AD. The association of *SORCS1* with both AD and T2D is sexually dimorphic in humans, with both disease associations showing more robust effects in females. Based on published evidence that manipulation of the mouse genome combining multiple genes related to cerebral amyloidosis, to T2D, or both, might provide novel mouse models with exacerbated amyloid and/or diabetes phenotypes, we assessed memory, glucose homeostasis, and brain biochemistry and pathology in male and female wild-type, *Sorcs1* -/-, *APP/PSEN1*, and *Sorcs1* -/- *X APP/PSEN1* mice.

**Results:**

Male mice with either the *APP/PSEN1* or *Sorcs1* -/- genotype displayed earlier onset and persistent impairment in both learning behavior and glucose homeostasis. Unlike prior examples in the literature, the behavioral and metabolic abnormalities in male mice were not significantly exacerbated when the two disease model mice (*Sorcs1* -/- models T2D; *APP/PSEN1* models AD*)* were crossed. However, female *Sorcs1* -/- *X APP/PSEN1* mice exhibited worse metabolic dysfunction than *Sorcs1* -/- knockout mice and worse memory than wild-type mice. The deletion of *Sorcs1* from *APP/PSEN1* mutant mice led to no obvious changes in brain levels of total or oligomeric amyloid-beta (Aβ) peptide.

**Conclusions:**

In general, unexpectedly, there was a trend for gene targeting of *Sorcs1-/-* to partially mitigate, not exacerbate, the metabolic and amyloid pathologies. These results indicate that crossing AD model mice and T2D model mice may not always cause exacerbation of both the amyloidosis phenotype and the metabolic phenotype and highlight the unexpected pitfalls of creating mixed models of disease.

**Electronic supplementary material:**

The online version of this article (doi:10.1186/s40478-016-0282-y) contains supplementary material, which is available to authorized users.

## Introduction

Alzheimer’s disease (AD) is a progressive neurodegenerative disorder and is the most common cause of dementia in the elderly. There are approximately 34 million people afflicted by AD worldwide and those numbers are expected to triple over the next 40 years [5]. Type 2 diabetes mellitus (T2D) increases the risk for dementia [15, 19, 42, 69]. Both T2D and dementia are highly complex and share several features, including impaired cognitive function, vascular dysfunction and oxidative and inflammatory stress [15, 54]. Several mechanisms have been proposed for this association, including hypercholesterolemia, vasculopathic factors, and insulin resistance [63]. Environmental factors, such as poor diet and obesity are also known to increase the predisposition to T2D and the risk for dementia [21]. The extent to which dementia is due to AD or due to vascular dementia is an area of great interest, and the pathology of dementia in T2D is usually mixed [1, 43, 65].

Rare, early-onset familial Alzheimer’s disease (EOFAD) is believed to begin with the accumulation of oligomeric forms of Aβ in the hippocampus and cortex. EOFAD is caused by mutations in genes that directly influence Aβ metabolism, most commonly the amyloid precursor protein (*APP*), presenilin1 (*PSEN1*) or presenillin 2 (*PSEN2*). Mice expressing pathogenic mutant forms of human *APP, PSEN*, or both, are mainstays of *in vivo* modeling of cerebral amyloidosis in AD research. Of note, mice harboring EOFAD-pathogenic mutations in both APP and PSEN1 have been observed to develop insulin resistance (Ruiz et al*. in press* [56]).

Genetic studies of late-onset Alzheimer’s disease (LOAD) point to a number of susceptibility genes, including several that belong to one of three classes of molecules: the apolipoprotein family (the most notable of which is apolipoprotein E [11, 59]; the low density lipoprotein receptor (LDLR) family [22, 31]; and the vacuolar protein sorting-10 (VPS10) domain containing receptor family. The AD-linked gene *SORL1* belongs to both the LDLR family and the VPS10-domain protein family, and a deficiency in SorL1 protein has been observed in the brains of patients suffering from LOAD [12, 33, 55, 58, 60]. SorL1 has been demonstrated to modulate Aβ generation via an interaction with the core component of the retromer complex, Vps35 [2, 39, 41, 61]. Human and animal studies in AD have implicated Vps35 and other components of the retromer complex [36, 64].

Multiple studies have demonstrated a connection between insulin resistance and T2D and increased risk for cognitive impairment in humans [4, 13, 34, 62]. *SORCS1*, another member of the Vps10 family, has been identified as a potential risk factor for LOAD and is decreased in AD brains [33]. *SORCS1* resides at a quantitative trait locus for T2D in mice and rats [10, 14] and has been associated with both T1D and T2D [13]. Previously, we initiated an investigation of the action of SorCS1 on APP metabolism. We observed that endogenous murine Aβ40 and 42 levels were increased in the brains of female but not male *Sorcs1* -/- mice, and this appeared to involve an interaction mediated by the Vps35 retromer complex [29]. This was especially interesting because the genetic linkage to *SORCS1* is sexually dimorphic with the linkage to women being more robust. We hypothesized that *SORCS1* hypomorphism might contribute to the link between insulin resistance and increased risk for cognitive impairment in humans. A corollary of that hypothesis is that dysregulation of SorCS1 may contribute to both the Aβ disturbance underlying AD and the insulin/glucose metabolism disturbance underlying DM, with the two genes arranged either in parallel or in series. Kebede et al. [23] demonstrated that *Sorcs1-/-* mice were not overtly diabetic unless they were crossed with leptin deficient (*ob/ob*) mice. In contemplating this result in the context of the AD/T2D association with *SORCS1*, this observation raised the possibility that *Sorcs1-/-* mice crossed with AD model mice (here, *APP/PSEN1* mice) might manifest overt diabetes if stressed by the accumulation of cerebral amyloid. Previous studies show that the combination of a genetic proamyloidogenic phenotype and potentially diabetogenic phenotype (induced by either drug, diet, or genetic manipulations) can lead to exacerbation of either the proamyloidogenic component of the phenotype, the prodiabetogenic component of the phenotype, or exacerbation of both components of the phenotype [8, 18, 20, 38, 46, 51, 52, 66].

Here we report characterization of the memory, glucose homeostasis and Aβ pathology in male and female wild-type, *Sorcs1* -/-, *APP/PSEN1* and *Sorcs1* -/- mice crossed with *APP/PSEN1* mice. Unexpectedly, there was a trend for gene targeting of *Sorcs1-/-* to mitigate, not exacerbate, the metabolic, behavioral, and cerebral amyloid angiopathic pathologies.

## Materials and methods

### Animals

Male and Female C57BL/6J wild-type (WT; *n* = 5–14/group), *Sorcs1* -/- (*n* = 10–16/group), *APP*^*Swe*^*x PSEN1*^*Δexon9*^ (*APP/PSEN1*; *n* = 5–11/group respectively) and *Sorcs1 -/- x APP*^*Swe*^*x PSEN1*^*Δexon9*^*(Sorcs1 -/- x APP/PSEN1; n* = 5–9/group) were bred in the Icahn School of Medicine at Mount Sinai Vivarium. We have previously described the generation of the *Sorcs1* -/- line [29]. All animal studies were conducted in accordance with National Institute of Health Guidelines for the Care and Use of Experimental Animals and approved by the Institutional Animal Care and Use Committee at the Icahn School of Medicine at Mount Sinai. Mice were kept in a pathogen-free environment on a 12-h light/dark cycle, and given *ad libitum* access to food and water. From weaning until 6 months of age, cohorts were maintained on standard rodent chow. Cohorts were subjected to learning behavior assessment at 5 and 9 mo of age. Cohorts were placed on a 60 % high fat diet (Diet 58Y1, TestDiet, St. Louis, MO) at 6 mo of age for 4–12 weeks. Mice were subjected to metabolic profiling prior to starting the HFD at 4–5 mo of age then again after 4–12 weeks of HFD. All mice were weighed weekly. Following final metabolic testing mice were anesthetized, and were then perfused with ice-cold PBS. The brain was removed and dissected into two hemispheres. One half was snap-frozen on dry ice and stored at -80 °C for Aβ biochemistry. The other half was post-fixed in 4 % PFA then cut into 30 μm coronal sections using a vibratome. Sections were stored in PBS with 0.1 % NaAz until histological analyses.

### Metabolic profiling

#### Body composition analyses

Total body fat and lean tissue were assessed using an EchoMRI quantitative magnetic resonance system (Echo Medical Systems) as previously described [57].

#### Glucose tolerance test (GTT)

Mice were fasted for 5 h in a quiet testing room with free access to water. Baseline fasted blood samples were then taken via tail vein bleed to measure plasma insulin, lipids, and branched-chain amino acids. Blood samples were collected into EDTA coated microvette tubes and placed on ice before being spun at 7 K for 6 min. Plasma was then removed, snap frozen on dry ice and stored at -80 °C until analyses. Mice were then injected intraperitoneally (i.p) with a 0.75 or 1.5 g/kg glucose solution (Sigma Aldrich, St. Louis, MO). Blood glucose was measured via tail vein bleed immediately before the glucose bolus then at 15, 30, 60, 90 and 120 min post injection.

#### Insulin tolerance test (ITT)

Mice were fasted for 5 h then given an i.p. injection of 1 or 2.5U/kg insulin (Humulin R, Lilly & Co., Indianapolis, IN) solution. Blood glucose levels were measured prior to and at 15, 30, 45, 60 and 90 min following the insulin bolus.

### Behavioral testing

Mice were placed in the testing room 1 h prior to testing to acclimatize to the room. All testing was completed between 8 am-4 pm. All equipment was cleaned between animals.

#### Novel object recognition (NOR)

Short-term non-associative memory based on the natural exploration of novelty in mice was assessed in the novel object recognition test as described [26]. Briefly, on day 1, the mouse was habituated in the NOR arena (20 cm diameter) for 10 min. On day 2, the mouse underwent the testing phase, composed of two stages. During testing phase 1, the mouse was placed in the arena and allowed to explore two identical unfamiliar objects for 10 min. The mouse was then returned to its home cage for an interval of 1 h. During this time, one of the two objects the mouse was previously allowed to explore was removed and replaced with a novel object. During testing phase 2, the mouse was placed back into the arena and allowed to explore the familiar object and the novel object for 4 min. Trials were videotaped using an overhead camera. The duration spent exploring the objects was then measured using ANY-maze (Stoelting, Wood Dale, IL). Exploration was defined as the amount of time the mouse spent pointing their nose within 2 cm of the object.

#### Y-maze spontaneous alternation (SA)

Short-term working was assessed in the Y-maze spontaneous alternation (SA) test as previously described [25] using a black opaque Perspex Y-maze with 3 arms (A, B, and C) each containing a visual cue (arm dimensions; 35x5x10 cm). Briefly, each animal was placed in turn in arm A of the Y-maze and allowed to explore for 8 min and the arm entries made by each animal were recorded. Arm entry was defined as having all 4 paws in the arm. Spontaneous alternation was defined as a successive entry into 3 different arms, on overlapping triplet sets. The percentage number of alternations was calculated as the number of actual alternations divided by the maximum number of alternations (the total number of arm entries minus 2).

### Biochemical and histological analyses

#### Plasma insulin, lipids, and branched-chain amino acid level determination

Baseline, 1 and 3 months post HFD, levels of plasma insulin were determined using a mouse insulin ELISA kit (Mercodia, Sweden) according to the manufacturer’s instruction. Glycerol and triglycerides were quantified using a plasma triglyceride assay (Sigma Aldrich, St. Louis, MO), which was modified to a 96-well plate format. Non-esterified fatty acid (NEFA) levels were determined using a NEFA kit from Wako (Wako Chemicals USA, Inc. Richmond, VA) following the manufacturer’s instructions. Plasma branched-chain amino acid (BCAA) levels were determined using a fluorimetric assay as described [6].

#### Aβ assay

Hemibrains were processed via differential detergent solubilization [40] to produce TBS-soluble, Triton-X-soluble and formic-acid soluble Aβ fractions. Total Aβ equals the sum of Aβ concentration in TBS-soluble, Triton X-soluble and formic acid-soluble fractions. For analysis of native oligomeric Aβ protein structure, 2–4 μl native protein samples (from the TBS-soluble fraction) were spotted onto activated/pre-wetted PVDF membrane (0.22 μm; Millipore) and allowed to dry. Following protein spotting, membranes were blocked for 1 h at room temperature in 5 % w/v non-fat milk (Santa Cruz) in TBS containing 0.1 % v/v Tween-20 (Fisher Scientific; TBS-T). Membranes were incubated in the indicated primary antibody (in 5 % milk/TBS-T) overnight at 4 °C, washed 4x in TBS-T, incubated in species-specific HRP-conjugated secondary antibody (in 5 % milk/TBS-T) for 1 h at room temperature, and then washed 4x in TBS-T. Membranes were subsequently developed with ECL Western blotting substrate (Pierce) using the Fujifilm LAS-3000 developer. Membranes were washed 1x in TBS-T and stripped in low pH stripping buffer [25 mM Glycine HCl, pH 2.0 and 1 % w/v SDS] to remove primary and secondary antibody, washed 3x in TBS-T, and blocked for 1 h (in 5 % milk/TBS-T) at room temperature before probing with the next primary antibody. Integrated density of immunoreactive spots was measured using MultiGauge Software (FujiFilm) and normalized to % control (vehicle). Generation, purification, and characterization of rabbit pAb A11 (anti-prefibrillar oligomers, 0.5 μg/ml), rabbit pAb OC (anti-fibrillar oligomers and fibrils; 0.25 μg/ml) and mouse mAb Nu-4 (anti-oligomers; 1 μg/ml) have been described previously [28, 67]. Normalization to total APP/Aβ signal was achieved by detection of human APP transgene metabolites with the mouse mAb 6E10 (1:1000; Covance). Peroxidase-conjugated goat anti-rabbit IgG (H + L; 1:20,000; Vector Labs) or goat anti-mouse IgG (H + L; 1:20,000; Vector Labs) were used for detection. To quantify total Aβ levels, human/rat Aβ 1–40/1–42 ELISA kits (Wako) were used according to the manufacturer’s instructions. Absolute concentrations of total or oligomeric Aβ were normalized to initial tissue weight prior to analysis.

#### Histology

Aβ was assessed via free-floating fluorescence immunohistochemistry using mAb 6E10 (1:1000, Covance, Princeton, NJ). To assess Aβ deposition within vessels sections were co-stained with collagen IV (1:300). Briefly, sections were washed 3x in PBS for 10 min, incubated in 3 % acetic acid for 10 min followed by 1 mg/ml Pepsin for 30 min. Following 1x in PBS/0.1 % NaAz for 10 min and 3x 10 min washes in PBS, sections were incubated 1 % H_2_O_2_ for 20 min, 3x further 10 min washes in PBS before blocking with 5 % goat serum in TBS/Triton-X for 1 h. Sections were then incubated with mAb 6E10 (1:1000, Covance, Princeton, NJ) and collagen-IV (1:300, Abcam ab6586) overnight at 4 °C. The following day, sections were given 3x 20 min washes in PBS then incubated with anti-mouse and anti-rabbit fluorescent secondary antibodies (1:200, mouse Alexa Fluor 488 and rabbit 594 respectively) at room temperature for 2 h. After 3x final 20 min washes in PBS, sections were mounted and coverslipped with Vectashield (Vector Laboratories, Inc., Burlingame, CA). Images were captured on an Olympus BX61 upright microscope with an attached Olympus DP71 camera.

Perls’s Berlin blue-stained clusters of hemosiderin staining were evaluated from sections throughout the neocortex, hippocampus, and thalamus. Briefly, sections were mounted on slides and dried overnight. The next days, slides were immersed in equal parts of 20 % hydrochloric acid and 10 % potassium ferrocyanide for 20 mins. Slides were then washed 3x in dH_2_0 then counterstained with nuclear fast red for 5 min. Following another 2x rinses in dH_2_0, sections were dehydrated through 95 % and 2x changes of 100 % ethanol. Sections were then cleared in xylene, coverslipped and allowed to dry overnight before imaging.

#### Statistical analyses

All data are presented as the mean ± s.e.m. Statistical significance (*P* < 0.05) was determined using Student’s *t* tests or one/two-way ANOVA with Bonferroni posthoc analyses (GraphPad Prism, San Diego, CA).

## Results

Glucose homeostasis and learning behavior were tested in mice at 4-5 mo of age. Young male and female WT, *Sorcs1* -/-, *APP/PSEN1* and *Sorcs1* -/- x *APP/PSEN1* mice had similar levels of fasting glucose and insulin (Additional file 1: Figure S1a-d) and normal ability to clear a glucose load when assessed by a GTT (Additional file 1: Figure S1a,c). Fasting plasma glycerol (Additional file 1: Figure S1i,k) and triglycerides (TG) (Additional file 1: Figure S1j,l) levels were unaltered indicating normal lipid homeostasis in mice of both sexes. Female *Sorcs1* -/- mice displayed lower body weight, shorter body length, and reduced lean mass (Additional file 1: Figure S1e,f,m) but comparable fat mass (Additional file 1: Figure S1n) when compared to WT mice. Female *Sorcs1* -/- mice were also reduced in weight and lean mass (Additional file 1: Figure S1e,m) as compared with *APP/PSEN1* mice. No differences in body weight or fat mass were seen among male cohorts (Additional file 1: Figure S1g,p). However, male *Sorcs1* -/- mice were shorter in length than *APP/PSEN*1 mice and *Sorcs1* -/- x *APP/PSEN1* mice had reduced lean mass as compared to WT mice (Additional file 1: Figure S1h,o). Crossing *Sorcs1* -/- with *APP/PSEN1* mice did not promote the onset of any metabolic phenotype in either female nor male mice.

**Fig. 1:**
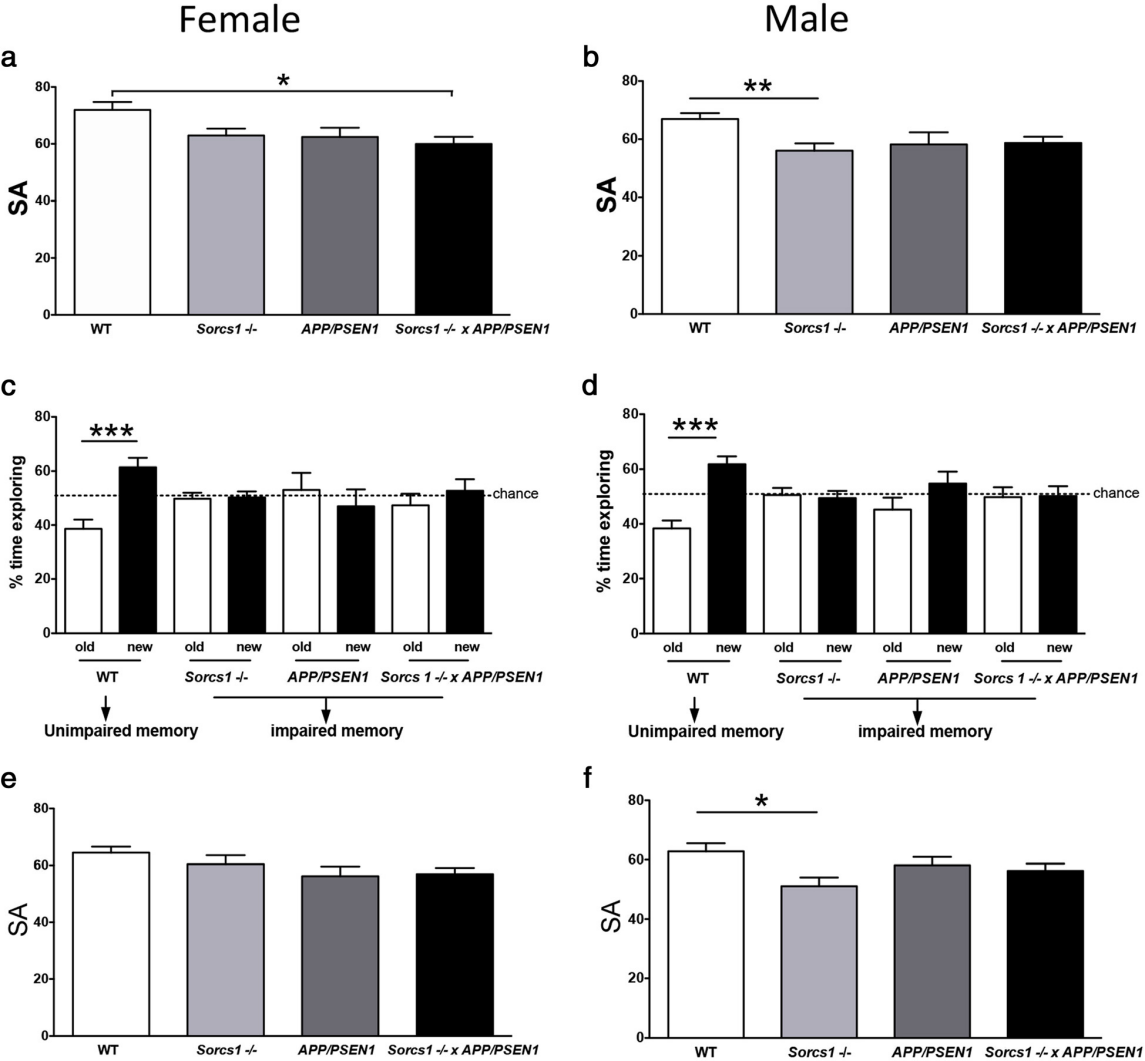
Female but not male *Sorcs1* -/- x *APP/PSEN1* mice display impaired memory. Spontaneous alternation (SA) was assessed at 5 mo (**a**, **b**) and re-tested again at 9 mo (**e**, **f**), while novel object recognition (NOR) was assessed at 5 mo (**c**, **d**) in cohorts of male and female WT (*n* = 11–14/group), *Sorcs1* -/- (*n* = 15–16/group), *APP/PSEN1* (*n* = 6–11/group) and *Sorcs1* -/- x *APP/PSEN1* (*n* = 9/group). One-way ANOVA with Bonferroni posthoc analyses; *P < 0.05, **P < 0.01 and ***P < 0.001. Data expressed as mean ± s.e.m

When behavior was assessed in Y-maze, both male and female young *Sorcs1 -/-*, *APP/PSEN1* and *Sorcs1 -/- x APP/*PSEN1 mice all had worse SA memory performance than WT mice, with only *Sorcs1 -/-* x *APP/PSEN1* reaching significance in the females and Sorcs1 -/- in the males (Fig. 1a-b). Memory was also assessed in the novel object recognition (NOR) test, both male and female WT mice had intact memory whereas male and female *Sorcs1* -/-, *APP/PSEN1*, and *Sorcs1* -/- x *APP/PSEN1* mice were all impaired and to a comparable degree (Fig. 1c-d).

**Fig. 2:**
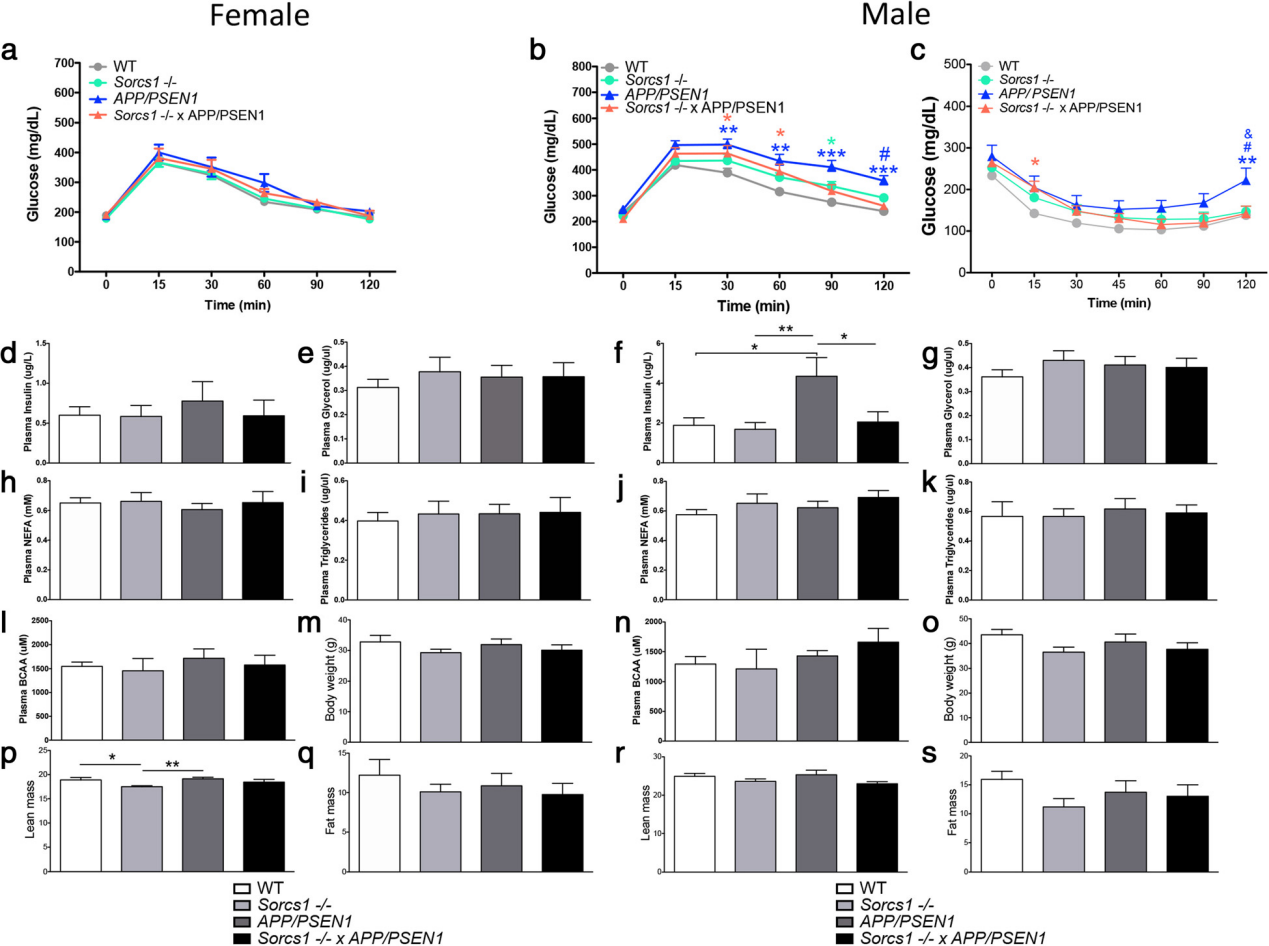
After 4 weeks high fat feeding, male but not female *APP/PSEN1*, *Sorcs1* -/- and *Sorcs1* -/- x *APP/PSEN1* mice demonstrate impaired glucose homeostasis compared to WT mice. Glucose tolerance testing (**a**-**b**), insulin tolerance testing (**c**) fasting plasma insulin (**d**, **f**), fasting plasma glycerol (**e**, **g**), fasting plasma non-esterified free fatty acids; NEFA (**h**, **j**), fasting plasma triglycerides (**i**, **k**), fasting plasma branched-chain amino acids; BCAA (**l**, **n**) body weight (**m**, **o**), lean mass (**p**, **r**) and fat mass (**q**, **s**) in cohorts of male and female WT (*n* = 5–12/group), *Sorcs1* -/- (*n* = 10–16/group), *APP/PSEN1* (*n* = 6–7/group) and *Sorcs1* -/- x *APP/PSEN1* (*n* = 4–9/group). Cohorts were maintained on standard rodent chow from weaning until 6 mo when they were placed on a 60 % high fat diet for 4 weeks. **a**-**c** two-way ANOVA with Bonferroni posthoc analyses; *P < 0.05, **P < 0.01 and ***P < 0.001 green = *Sorcs1 -/- versus* WT, blue = *APP/PSEN1* versus WT, red = *Sorcs1 -/-* x *APP/PSEN1* versus WT, ^#^ *P* < 0.05 blue = *APP/PSEN1 versus Sorcs1 -/- x APP/PSEN1*, ^&^ *P* < 0.05 blue = *Sorcs1* -/- versus *APP/PSEN1*. **d**-**s** one-way ANOVA with Bonferroni posthoc analyses; *P < 0.05 and **P < 0.01. Data expressed as mean ± s.e.m

In order to determine whether cohorts in this study were susceptible to the induction of metabolic dysregulation by high fat feeding, a commonly used metabolic stressor to unveil a metabolic phenotype, all mice were placed on a 60 % high fat diet (HFD) for 4 weeks. All female mice were resistant to diet-induced perturbations in glucose homeostasis (Fig. 2a) with no genotype-dependent differences in fasting plasma metabolic parameters detected at 7 mo of age, consistent with the notion that female sex is protective against a metabolic phenotype (Fig. 2d-e, h-I,l). After 4 weeks of HFD feeding, differences in body weight observed on regular chow were no longer statistically significant (Fig. 2m). As noted above for regular chow feeding, female *Sorcs1* -/- mice on HFD continued to have less lean mass than WT or *APP/PSEN1* mice (Fig. 2p). While all mice gained a significant amount of fat mass on HFD, body composition analysis revealed no differences among cohorts (Fig. 2q).

After 4 weeks on HFD and in comparison to WT mice at baseline (4-5 mo of age), male *APP/PSEN1*, *Sorcs1* -/-, and *Sorcs1* -/- x *APP/PSEN1* mice displayed impaired glucose tolerance (Fig. 2b) and *APP/PSEN1* and *Sorcs1* -/- x *APP/PSEN1* mice impaired insulin sensitivity (Fig. 2c). Unexpectedly, *APP/PSEN1* male had worse glucose tolerance and insulin sensitivity than *Sorcs1* -/- x *APP/PSEN1* mice (Fig. 2b-c). Insulin sensitivity was also worse in *APP/PSEN1* mice than in *Sorcs1* -/- mice (Fig. 2c). This was also reflected in higher levels of fasting plasma insulin in male *APP/PSEN1* mice compared to all other groups (Fig. 2f). All other metabolic parameters were unaltered (Fig. 2g, j-k, n) as was body weight (Fig. 2o) and lean mass (Fig. 2r). As with females, all males gained a significant amount of fat mass on the HFD as seen by body composition analyses (Fig. 2s). These data are consistent with enhanced susceptibility to HFD induced metabolic dysregulation of *APP/PSEN1* mice (Ruiz et al., in press [56]).

**Fig. 3:**
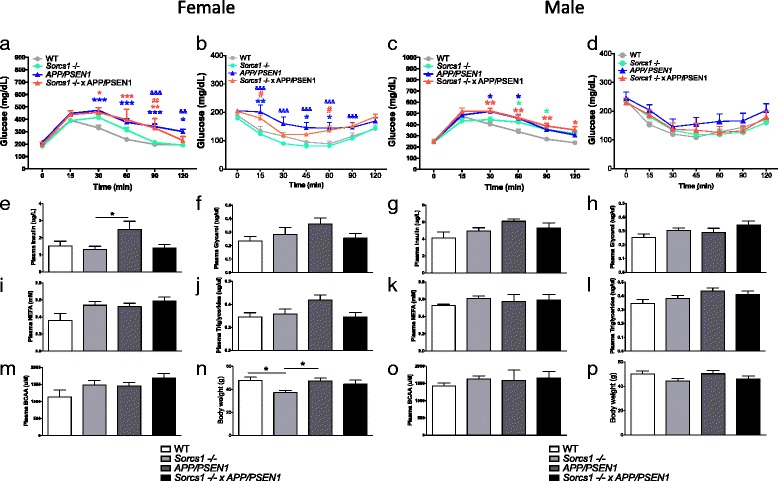
After 12 weeks high fat feeding, female *Sorcs1* -/- x *APP/PSEN1* mice demonstrate greater impairment in glucose intolerance and insulin sensitivity than *Sorcs1* -/- mice. Glucose tolerance testing (**a**, **c**), insulin tolerance testing (**b**, **d**) fasting plasma insulin (**e**, **g**), fasting plasma glycerol (**f**, **h**), fasting plasma non-esterified free fatty acids; NEFA (**i**, **k**), fasting plasma triglycerides (**j**, **l**), branched-chain amino acids; BCAAs (**m**, **o**) and body weight (**n**, **p**) in cohorts of male and Female WT (*n* = 5–12/group), *Sorcs1* -/- (*n* = 10–16/group), *APP/PSEN1* (*n* = 6–7/group) and *Sorcs1* -/- x *APP/PSEN1* (*n* = 4–9/group). Cohorts were maintained on standard rodent chow from weaning until 6 mo when they were placed on a 60 % high fat diet for 12 weeks. **a**-**d** two-way ANOVA with Bonferroni posthoc analyses; *P < 0.05, **P < 0.01 and ***P < 0.001 green = *Sorcs1 -/- versus* WT, blue = *APP/PSEN1* versus WT, red = *Sorcs1 -/-* x *APP/PSEN1* versus WT, ^#^ *P* < 0.05 and ^##^ *P* < 0.01 red = *Sorcs1 -/- versus Sorcs1 -/- x APP/PSEN1*, ^&&^ *P* < 0.01 and ^&&&^ *P* < 0.001 blue = *Sorcs1* -/- versus *APP/PSEN1*. **e**-**p** one-way ANOVA with Bonferroni posthoc analyses; *P < 0.05. Data expressed as mean ± s.e.m

Aging is a risk factor for both AD and T2D. To determine whether cohorts were more susceptible to aging-induced insulin resistance and/or glucose intolerance, mice were maintained on the HFD for an additional 8 weeks i.e., to a total of 12 weeks on HFD. During this extension of HDF feeding, abnormalities in glucose homeostasis emerged in females cohorts. Both female *APP/PSEN1* and *Sorcs1* -/- x *APP/PSEN1* mice displayed impaired glucose tolerance (Fig. 3a), and *APP/PSEN1* mice displayed impaired insulin sensitivity (Fig. 3b). The metabolic phenotype was more severe in female *APP/PSEN1* and *Sorcs1* -/- x *APP/PSEN1* mice than in female *Sorcs1* -/- mice (Fig. 3a-b). After 12 weeks of HFD, fasting plasma insulin levels were elevated in all genotype groups but this elevation only reached statistical significance in female *APP/PSEN1* mice (Fig. 3e). All other metabolic parameters were unaltered (Fig. 3f, i, j, m) across other cohorts. *Sorcs1 -/-* mice had lower body weight than WT and *APP/PSEN1* mice (Fig. 3n).

Male *APP/PSEN1*, *Sorcs1* -/-, and *Sorcs1* -/- x *APP/PSEN1* mice continued to display impaired glucose tolerance (Fig. 3c) compared to WT mice. Interestingly, the reduced insulin sensitivity in male mice observed after 4 weeks HFD and noted above was less severe after 12 weeks of HFD (Fig. 3d). In all groups, maintenance on HFD was associated with increased fasting plasma insulin levels; however, the elevated plasma insulin level previously observed in male *APP/PSEN1* mice was no longer obvious because the plasma insulin levels in other groups were now similarly increased (Fig. 3g). All other metabolic parameters were unaltered (Fig. 3h, k-l, o) as was body weight (Fig. 3p) when compared across other cohorts.

When learning behavior was re-tested after 12 weeks on HFD, all groups of female mice (regardless of WT vs genetically manipulated) displayed similar performance (Fig. 1e). As observed at baseline and as mentioned above, male *Sorcs1* -/- mice had poorer performance than WT mice in SA memory (Fig. 1f). No other groups of male mice displayed learning behavior deficits that reached statistical significance.

**Fig. 4:**
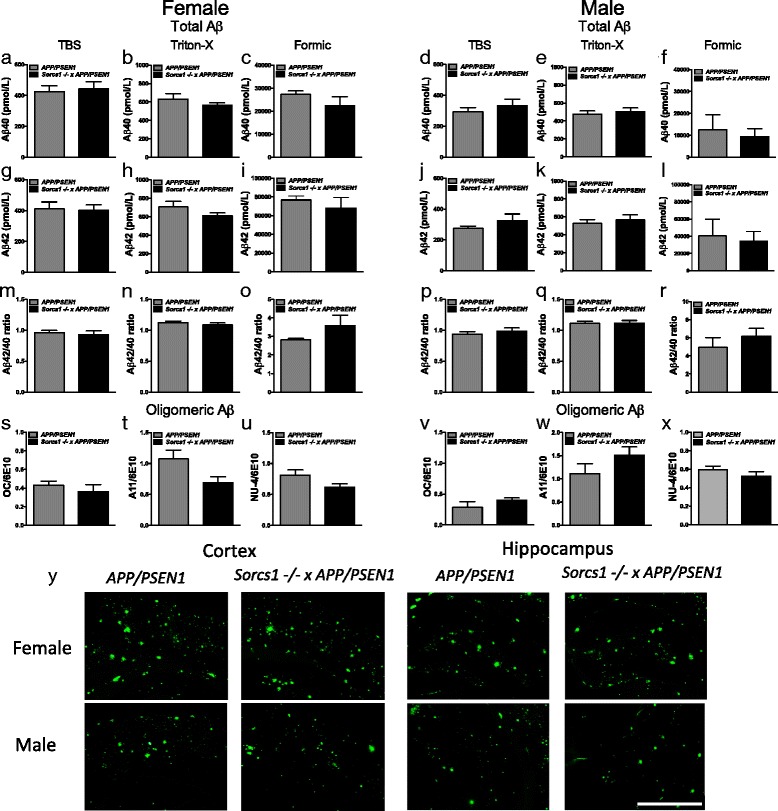
*APP/PSEN1* and *Sorcs1* -/- x *APP/PSEN1* have similar levels of Aβ as well as plaque load within the brain. Hemibrains of male and female *APP/PSEN1* (*n* = 5–12/group) and *Sorcs1* -/- x *APP/PSEN1* (*n* = 10/group) mice were processed via differential detergent solubilization to produce fractions of TBS-soluble, Triton-X-soluble and formic-acid soluble Aβ. Levels of Aβ 40 (**a**-**f**) and Aβ 42 (**g**-**l**) were determined from each fraction via ELISA. Total Aβ equals the sum of Aβ from each fraction. The Aβ42/40 ratio was calculated for each fraction (**m**-**r**). Oligomeric Aβ was assessed from the TBS-soluble fraction via dotblot analyses using OC (**s**, **v**), A11 (**t**, **w**) and NU-4 (**u**, **x**) antibodies. 6E10 positive Aβ plaque load was assessed via immunohistochemistry within the cortex and hippocampus (**y**) of male and female *APP/PSEN1* and *Sorcs1* -/- x *APP/PSEN1* mice (*n* = 2-3/group). ***a*** *-* ***x*** *P* > 0.05, Student’s t-tests. Data expressed as mean ± s.e.m. **y** scale bar = 1 mm

**Fig. 5:**
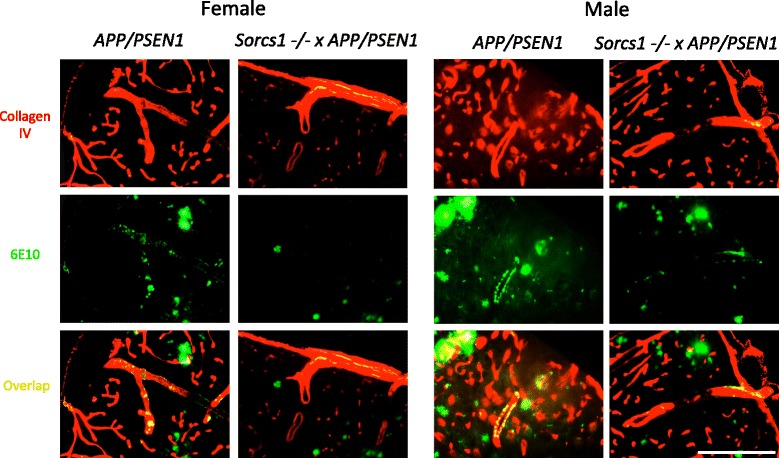
*APP/PSEN1* and *Sorcs1* -/- x *APP/PSEN1* mice display vascular Aβ within the brain. Aβ was detectible within cerebral blood vessels of male and female *APP/PSEN1* and *Sorcs1* -/- x *APP/PSEN1* mice (*n* = 2–3/group) when assessed via immunohistochemistry using 6E10 and collagen IV. Data expressed as mean ± s.e.m. Scale bar = 200 μm

**Fig. 6:**
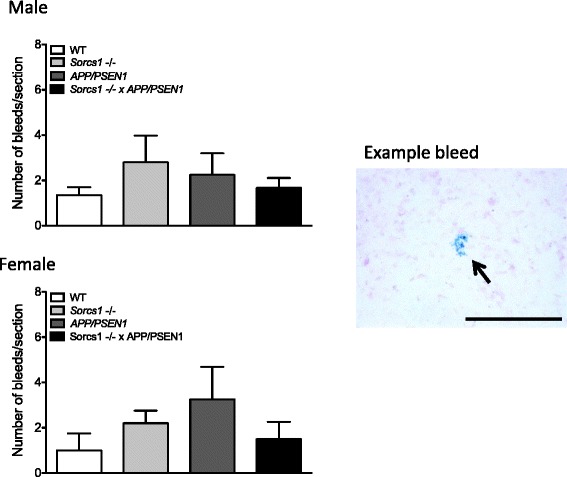
*APP/PSEN1* and *Sorcs1* -/- x *APP/PSEN1* mice display micro-hemorrhages within the brain. Perl’s blue positive micro-hemorrhages were seen within the brains of cohorts of all male and female WT, *Sorcs1* -/-, *APP/PSEN1* and *Sorcs1* -/- x *APP/PSEN1* (*n* = 2–4/group) when assessed via immunohistochemisty. *P* > 0.05, Student’s t-tests. Data expressed as mean ± s.e.m. Scale bar = 200 μm

After 12 weeks of HFD feeding, mice were sacrificed to determine whether *Sorcs1* deficiency exacerbated brain Aβ accumulation. *Sorcs1* -/- x *APP/PSEN1* mice displayed similar levels of total brain Aβ40 (Fig. 4a-f), Aβ42 (Fig. 4 g-l), Aβ42/40 ratio (Fig. 4m-r) and Aβ oligomers (Fig. 4s-x) compared to *APP/PSEN1* mice in both male and female cohorts. Similarly, no differences in Aβ plaque load were detected between the two genotypes (Fig. 4y). Notably, females had much higher levels of insoluble Aβ in the formic acid fraction than males. As expected due to the absence of human Aβ, no plaques were observed in either male or female WT and *Sorcs1* -/- mice (data not shown). Interestingly, Aβ was deposited as cerebral amyloid angiopathy (CAA) in large and small blood vessels within the brains of male and female *APP/PSEN1* mice as well as *Sorcs1* -/- x *APP/PSEN1* mice (Fig. 5). Quantification of CAA is challenging at best, but, at the qualitative level, male and female *Sorcs1* -/- x *APP/PSEN1* mice appeared to have less vascular Aβ than *APP/PSEN1* mice and when present was confined to large vessels (Fig. 5). Microhemorrhages were also detectable within the brains of all cohorts (Fig. 6). As observed with the CAA, the brains of both male and female *APP/PSEN1* mice appeared to display more cerebral microhemorrhages than WT mice, and the brain microhemorrhage count was less in *Sorcs1 -/-* X *APP/PSEN1* mice than in *APP/PSEN1* mice. Unexpectedly, the microhemorrhage count in *Sorcs1 -/-* mice was similar to that of *Sorcs1 -/-* X *APP/PSEN1* mice despite the absence of human Aβ or CAA in *Sorcs1 -/-* mice.

## Discussion

Multiple epidemiological studies have demonstrated an association between insulin resistance and T2D and increased risk for cognitive impairment in humans [4, 13, 34, 62]. *SORCS1* has been associated with both T2D and AD. Furthermore, the human linkage to *SORCS1* is stronger for women in both T2D and AD. Here, we tested the hypothesis that a mouse model with a compound proamyloidogenic/prodiabetogentic genotype might be useful in studying how the two conditions coexist with possible bidirectional exacerbation (i.e., that cerebral amyloidosis exacerbated glucose intolerance and vice versa). In order to test this hypothesis, we assessed longitudinal aging-related memory behavior, glucose homeostasis, and Aβ pathology in both male and female WT, *Sorcs1* -/-, *APP/PSEN1* and *Sorcs1* -/- mice crossed with *APP/PSEN1* mice.

In the present study we demonstrate that when young, both male and female *Sorcs1* -/-, *APP/PSEN1* and *Sorcs1* -/- x *APP/PSEN1* mice display normal glucose homeostasis. This fits with previous studies that have shown that young *APP/PSEN1* mice have similar glucose tolerance to WT mice when fed a standard diet [17]. At this age, crossing *Sorcs1* -/- mice with *APP/PSEN1* did not precipitate earlier onset of metabolic dysfunction in either males or females. Interestingly, young female *Sorcs1* -/- displayed lower body weight, shorter body length and reduced lean mass when compared to WT mice. Female *Sorcs1* -/- mice were also shorter in length than *APP/PSEN1 and Sorcs1* -/- x *APP/PSEN1* had reduced lean mass compared to WT mice. This is the first study to report such difference and the cause is unknown. Lean mass is important for insulin action and metabolic control in that it has salutary effects. If two mouse models have the same metabolic flexibility but one has a higher lean mass, one can argue that the beneficial effects of higher lean mass could offset disturbances in metabolic flexibility.

When behavior was assessed in the Y-maze, the *Sorcs1* -/- mice X *APP/PSEN1* cross was associated with significantly impaired learning behavior in the females and trend for worse performance in *Sorcs1 -/-* and *APP/PSEN1* mice, although this did not reach statistical significance. In males, all cohorts had a worse performance in the Y-maze than WT mice but only *Sorcs1* -/- were significantly impaired. When behavior was re-assessed in the females, cohorts were no longer significantly different, the reason for this appears to be the worsening of memory in the WT group, the reason for this is unknown but could possibly due to the high fat diet, or age related decline, rather any change in the other cohorts which were already poor. In the male cohort, the *Sorcs1 -/-* mice continue to be impaired when compared to WT mice. When NOR was assessed, both male and female WT mice displayed intact learning behavior whereas male and female mice of *Sorcs1* -/-, *APP/PSEN1* and *Sorcs1* -/- x *APP/PSEN1* genotypes were all impaired. This is the first study to demonstrate learning deficits in *Sorcs1* -/- or *Sorcs1* -/- x *APP/PSEN1* mice. Unexpectedly in the present study both male and female *APP/PSEN1* mice and *Sorcs1* -/- mice displayed very early onset learning behavior deficits in NOR. For this reason, the NOR test was not repeated at later time points. Overall, the behavioral data for the Y-maze and NOR for both genders trend in the same direction for the most part. We cannot discount that lack of significance between some groups may be due to lack of power or sensitivity of these tests at the time points tested. Additional studies are required to dissect the temporal onset of NOR deficits in the *Sorcs1* -/- x *APP/PSEN1* mice and any potential sex differences in Y-maze.

In order to deliver a metabolic stress to unveil a metabolic phenotype, *APP/PSEN1* mice have previously been placed on either a high fat diet or crossed with transgenic models of obesity and/or diabetes. When we placed mice on a 60 % HFD, we found that male *APP/PSEN1*, display evidence of transient elevated fasted plasma insulin as well as both glucose intolerance and insulin insensitivity. During the development of T2D, β-cells initially compensate for insulin resistance by increasing the amount of insulin in circulation. This is consistent with previous studies where *APP/PSEN1* mice have increased fasted insulin, develop glucose intolerance and become less insulin sensitive when placed on a HFD [9, 35, 52, 70]. These results demonstrate that *APP/PSEN1* mice are susceptible to metabolic impairment on a HFD, consistent with a predisposition for the development of diabetes. Male *Sorcs1* -/- and *Sorcs1* -/- x *APP/PSEN1* displayed impaired glucose tolerance when compared to WT mice. Unexpectedly, metabolic abnormalities in male *Sorcs1* -/- mice were not exacerbated when crossing this line with the *APP/PSEN1* mouse. Indeed, there was a clear trend for *Sorcs1* deficiency to mitigate *APP/PSEN1* phenotypes. In general, the metabolic phenotype in the *Sorcs1* -/- line was much milder than expected, especially when compared to the *APP/PSEN1* line.

The female mice in the present study were resistant to metabolic abnormalities when placed on the diet for only 4 weeks. It is well known that female mice are resistant to metabolic effects of a high fat diet, which may account for their delayed onset of metabolic dysfunction when compared to males here. Petterson et al*.* [44] showed that in response to a high fat diet, plain C57Bl/6 male but not female mice developed hyperinsulinemia, hypotrophic islets, low grade systemic inflammation and an increased macrophage population in their intra-abdominal adipose tissue. In contrast, female mice had an elevated anti-inflammatory regulatory T cell population in their adipose tissue [44]. In the present study, after 12 weeks of HFD, female *APP/PSEN1* mice displayed metabolic abnormalities and increased fasted plasma insulin levels. Both female *APP/PSEN1* and *Sorcs1* -/- x *APP/PSEN1* mice had impaired glucose tolerance and *APP/PSEN1* mice had impaired insulin sensitivity. Unlike males, the female *Sorcs1* -/- x *APP/PSEN1* had significantly worse glucose tolerance and insulin sensitivity than female *Sorcs1* -/- mice. The lack of deficit in glucose tolerance in males between *Sorcs1* -/- x *APP/PSEN1* and *Sorcs1 -/-* is most likely due to the latter showing a significant deficit less visible in the females.

While *APP/PSEN1* overexpression facilitated glucose dysregulation, despite differences in glucose metabolism seen between *Sorcs1 -/-* and *Sorcs1* -/- x *APP/PSEN1* after 12 weeks of HFD, unexpectedly, *Sorcs1 -/-* crossed onto *APP/PSEN1* mice did not modulate brain Aβ levels or oligomer levels beyond the changes already induced by the *APP* and *PSEN1* mutations. The unexpected lack of worsening of pathology in the *Sorcs1* -/- x *APP/PSEN1* mice may indicate that Aβ deposition may be the cause rather than the effect of alterations in glucose metabolic observed. In support of this formulation, we have previously described the appearance of metabolic inflexibility in *APP/PSEN1* mice following high fat feeding (Ruiz et al*.*, in press [56]). In the present study, Aβ levels were measured from whole forebrain extracts; therefore, we cannot exclude possible subtle changes in regional Aβ levels relevant to memory behavior (such as the hippocampus and entorhinal cortex) and/or regulation of energy balance (the hypothalamus). Future studies would need to pick apart regional Aβ response and hypothalamic insulin signaling in *Sorcs1 -/-* and *Sorcs1 -/-* x *APP/PSEN1* mice. We cannot also exclude potential subtle changes in endogenous murine Aβ levels as have previously shown that female *Sorcs1 -/-* mice have elevated endogenous murine levels compared to WT mice [29]. Previously, Reitz et al*.* [53] reported that genetic variants in *SORCS1* are associated with increased risk of AD and that over-expression of SorCS1 reduces Aβ levels and γ-secretase activity, whereas suppression of SorCS1 increases Aβ levels and APP processing by γ-secretase activity. Unexpectedly, however, ablation of *Sorcs1* in the present study impacted memory without affecting production/accumulation of whole brain levels of Aβ. Although we can’t discount subtle altered levels of endogenous murine Aβ in *Sorcs1* -/- mice as we have previously reported [29] nor changes in regional Aβ as discussed above, there may be other more generalized effects that *SORCS1* may have on memory and neurodegeneration. Prominent neuronal expression of the Vps10p-domain sorting receptor family, of which SORCS1 is a member, has resulted in recent data supporting their role in neuronal activity, plasticity related processes and neurogenesis [53]. Furthermore, several studies have demonstrated links between SNPs in *SORCS1* and cognition. Printy et al. [49] assessed atrophy profiles against genetic markers in the Alzheimer’s Disease Neuroimaging Initiative (ADNI) cohort, finding associations of cerebral atrophy with SNPs on APP but also ventricular enlargement with SNPs on *SORCS1*. Reitz et al. [53] also assessed the impact of genetic variation in *SORCS1* and memory retention and found three SNPS were associated with memory retention.

Studies in other systems examining the relationship between AD pathology and diabetes have yielded variable results. One study revealed no association of the two pathologies [16]. In another study, cerebral amyloidosis was only exacerbated in diabetics carrying the *APOE ε4* allele [43], while in another, the result was that AD pathology was reduced among diabetics [7]. Interestingly, one of these studies also showed that the combined effects of carrying the ε4 allele and being diabetic increased the risk for a dementia that was clinically classified as vascular dementia [43]. Previous studies have also shown an association between both diabetes and vascular dementia [27, 30, 42]. More recently, Arvanitakis et al*.* [3] found that diabetes was associated with increased risk of brain infarction but not AD pathology in older individuals. At a recent workshop on AD and T2D at the US National Institutes of Health, the consensus was that the clinical cognitive decline in diabetics was probably more attributable to vascular dementia (Stoeckel, Gandy, and Arvanitakis, manuscript in preparation).

In the present study we assessed vascular Aβ, finding Aβ in blood vessels within the brains of both male and female *APP/PSEN1* mice as well as *Sorcs1* -/- x *APP/PSEN1* mice. Qualitative assessment revealed that both male and female *APP/PSEN1* mice displayed abundant deposits of Aβ in both large and small caliber vessels. Female *Sorcs1* -/- x *APP/PSEN1* mice appeared to have less vascular Aβ than *APP/PSEN1* mice and, when present, CAA was restricted to large caliber vessels. Male *Sorcs1* -/- x *APP/PSEN1* appeared to display relatively less vascular Aβ, and, again when present was restricted to large vessels. It is possible that reduced CAA could be a marker of altered Aβ trafficking and/or clearance, which should be an area of interest in future study. We also assessed microhemorrhages, and bleeds were detected in all mice assessed after HFD. Microhemorrhage in the WT mice is consistent with increased risk of hemorrhagic strokes associated with poor diet and obesity [45]. Both male and female *APP/PSEN1* mice tended toward greater numbers of bleeds than WT mice. This is consistent with previous studies that have shown that *APP/PSEN1* mice are prone to vascular alterations, which are exacerbated on a HFD [52]. Both male and female *Sorcs1* -/- mice revealed more bleeds than WT mice.

The extent to which dementia in humans is due to AD rather than to vascular dementia is an area of great interest, and the pathology of dementia in T2D is usually mixed [1, 43]. Human studies have yielded a range of results, with some, but not all, linking cerebral amyloidosis to insulin resistance [68]. Mouse models have been similarly variable. When APP mice are crossed with the leptin-deficient *ob/ob* model they showed no change in Aβ burden, however both amyloid angiopathy and angiitis were increased together with exacerbated cognitive dysfunction [66], while in contrast when Tg2576 mice are crossed with Irs2-/- insulin resistant mice show reduced Aβ pathology and cognitive improvement [24]. Recently, Ramos-Rodriguez et al*.* [51] crossed *APP/PSEN1* mice with the with the morbidly obese and diabetic *db/db* model, finding fewer plaques, a shift in soluble/insoluble pool of Aβ, increased microglia activation and increased hemorrhage burden in the crossed mice. Niedowicz et al*.* [38] also crossed *APP/PSEN1* mice with *db/db* mice, finding that the crossed mice displayed extreme obesity, diabetes and parenchymal Aβ deposition with strikingly severe cerebrovascular pathology of aneurysms and small strokes. As with the present study, the crossed mice in the Niedowicz study did not develop elevated levels of Aβ deposition. The crossed mice had more impairment in learning behavior in the Morris water maze than *db/db or APP/PSEN1* alone. Neither the crosses of Ramos-Rodriguez [51] nor those of Niedowics [38] predicted the results that we observed wherein the diabetogenic mutant mitigated the phenotype of the AD model.

Previous studies in diabetic models have documented increased angiogenesis and arteriogenesis, consisting of immature, unstable blood vessels leading to increased permeability of the blood–brain barrier [32, 38, 47, 48]. In the present study, we found evidence of both vascular Aβ and microhemorrhages, however, *Sorcs1* -/- x *APP/PSEN1* mice did not display an exacerbated phenotype; if anything, there was a trend toward reduced vascular Aβ and hemorrhage load in the *Sorcs1* -/- x *APP/PSEN1* mice when compared with the *APP/PSEN1* mice. Similarly, a recent study in which diabetes was induced in *APP/PSEN1* mice using streptozotocin treatment documented reduced amyloid angiopathy when compared to sham controls. This was associated with a slight reduction in plaque load and increased microglial activation [50]. In the case of the genes and models that we have studied, we tentatively conclude that metabolic abnormalities in female *Sorcs1* -/- x *APP/PSEN1* mice are not linked to changes in Aβ metabolism nor in brain bleeds; however, further detailed quantitative investigation of the vasculature in this model would be required to establish this proposed conclusion. The role of inflammation in the *Sorcs1* -/- x *APP/PSEN1* mice also requires further investigation.

In accordance with the present study, Kebede et al. [23] assessed the metabolic profile of female *Sorcs1* -/- mice over time, finding that without intervention they do not develop diabetes. Glucose intolerance was apparent in *Sorcs1 -/-* mice tested at 20 weeks of age. As with the present study, no mutation-related differences in fasted plasma insulin were observed up to 18 weeks. No abnormality in insulin sensitivity was detected at 12 weeks. The authors went on to place the *Sorcs1* -/- mice on a HFD, and, while they saw trends toward elevated glucose levels in response to a GTT, the trends were not significant. When Kebede et al*.* [23] crossed the female *Sorcs1* -/- mice with the leptin-deficient model of obesity (*ob/ob*), the crossed mice went on to develop diabetes, with elevated levels of fasting glucose and insulin as well as impaired glucose tolerance and sensitivity. On top of a diabetic phenotype, *Sorcs1* deficiency in the *ob/ob* model lead to severe depletion of insulin granules in pancreatic β-cells, leading the authors to suggest that *Sorcs1* is involved in vesicular trafficking and possibly biogenesis of insulin granules. As with the present study, the metabolic phenotype of *Sorcs1* -/- mice as characterized by Kebede et al*.* [23] required genetic or diet-induced obesity to be triggered. Therefore, as with the unexpectedly mild metabolic and amyloidosis phenotypes of *Sorcs1* -/- x *APP/PSEN1* mice as opposed to the exacerbated phenotypes of other combined diabetic/cerebral amyloidotic mouse models, the *APP/PSEN1* transgenes were apparently less stressful to beta cells and/or insulin action when compared to the metabolic stress induced by *db/db*. Previous studies show that the combination of a genetic proamyloidogenic phenotype and potentially diabetogenic phenotype (induced by either drug, diet, or genetic manipulations) can lead to exacerbation of either the proamyloidogenic component of the phenotype, the prodiabetogenic component of the phenotype, or exacerbation of both components of the phenotype [8, 18, 20, 38, 46, 51, 52, 66]. Unexpectedly, not only did the two genetic lesions not exacerbate each other in the present study, the *Sorcs1* deficiency tended to partially correct the diabetogenic phenotype of the *APP/PSEN1* mice. It is worth noting that “bidirectional exacerbation” is not universally observed, since Murakami et al*.* [37] reported that insulin receptor mutation-induced insulin resistance failed to exacerbate an Alzheimer’s-like phenotype in mice. Thus, while our experience is in the minority, our observations of no bidirectional exacerbation are not singular.

Kebede et al*.* [23] have demonstrated that stressing *Sorcs1-/-* mice by crossing with *db/db* is required to reveal the islet failure phenotype. The formulation for the basis of that model is that missorting and misprocessing of insulin constitutes the underlying pathogenesis. Our data would suggest that the *APP/PSEN1* proamyloidogenic phenotype is not sufficiently metabolically stressful to cause islet failure. This is our qualitative explanation for the non-exacerbation of the metabolic phenotype. Our formulation for the mechanism by which *Sorcs1-/-* modulates Aβ generation involves retromer dysfunction and promotion of generation of the pool of Aβ that arises from wildtype APP. For wildtype APP, 75 % of secreted Aβ is generated by endosomes and 25 % is generated in the trans Golgi network (TGN). However, when the substrate is Swedish mutant APP (i.e., APPKM670/671NL), the compartmental stoichiometry is altered, such that 75 % of secreted Aβ derived from Swedish APP is generated in the TGN and only 25 % is generated in the endosome. We would propose that *Sorcs1-/-*, by disrupting protein sorting in the TGN-endosomal system, reduces access of TGN BACE (β-APP sorting enzyme) to Swedish APP, thereby partially reversing the proamyloidogenic phenotype.

## Conclusions

In the present study we assessed memory, glucose homeostasis, and brain biochemistry and pathology in male and female WT, *Sorcs1* -/-, *APP/PSEN1*, and *Sorcs1* -/- *X APP/PSEN1* mice. Male mice with either the *APP/PSEN1* or *Sorcs1* -/- genotype displayed earlier onset and persistent impairment in both glucose homeostasis and in learning behavior. Unlike prior examples in the literature, the behavioral and metabolic abnormalities in male mice were not exacerbated when the two disease model mice (*Sorcs1* -/- models T2D; *APP/PSEN1* models AD*)* were crossed. In contrast to the male *Sorcs1* -/- *X APP/PSEN1* mice, female *Sorcs1* -/- *X APP/PSEN1* mice showed later onset but worse metabolic dysfunction than *Sorcs1* -/- knockout mice and worse memory than WT mice. The deletion of Sorcs1 from *APP/PSEN1* mutant mice led to no obvious changes in brain levels of total or oligomeric Aβ peptide. In general, unexpectedly, there was a trend for gene targeting of *Sorcs1-/-* to mitigate, not exacerbate, the metabolic, behavioral, and amyloid pathologies. Mixed pathology is a feature of some -- perhaps most -- late life dementia, raising the likelihood that compound genotypes may be required in order to approximate the pathology in mice, these results indicate that compound genotypes of highly complex diseases (such as AD plus T2D) may yield unexpected phenotypes that limit their utility.

## Additional file

Additional file 1: Figure S1. Young *Sorcs1 -/-*, *APP/PSEN1* mice and *Sorcs1 -/-* x *APP/PSEN1* mice display normal glucose homeostasis. Glucose tolerance testing (a,c), fasting plasma insulin (b,d), body weight (e,g), body length (f,h), fasting plasma glycerol (i,k), fasting plasma triglycerides (j,l), lean mass (m,o) and fat mass (n,p) in cohorts of male and female WT (*n* = 5- 8/group), *Sorcs1* -/- (*n* = 10/group), *APP/PSEN1* (*n* = 4-8/group) and *Sorcs1 -/-* x *APP/PSEN1* (*n* = 4- 6/group). Cohorts were maintained on standard rodent chow from weaning and assessed at 4-5 months of age. **P* < 0.05, ***P* < 0.01 and ****P* < 0.001; One-way ANOVA with Bonferroni posthoc analyses. Data expressed as mean ± s.e.m. (PDF 350 kb)

## Abbreviations

AD: Alzheimer’s disease
Aβ: Amyloid beta
APP: Amyloid precursor protein
BCAA: Branched chain amino acids
DM: diabetes mellitus
EOFAD: Early-onset familial Alzheimer’s disease
GWAS: Genome-wide association study
GTT: Glucose tolerance test
HFD: High fat diet
ITT: Insulin tolerance test
i.p: Intraperitoneally
LOAD: Late-onset Alzheimer’s disease
LDLR: Low density lipoprotein receptor
NEFA: Non-esterified fatty acids
NOR: Novel object recognition
PSEN1: Presenilin 1
PSEN2: Presenilin 2
SA: Y-maze spontaneous alternation
TG: Triglycerides
T2D: Type 2 diabetes
VPS10: Vacuolar protein sorting-10
WT: Wild-type

## References

[1] Ahtiluoto S, Polvikoski T, Peltonen M, Solomon A, Tuomilehto J, Winblad B (2010). Diabetes, Alzheimer disease, and vascular dementia: a population-based neuropathologic study. Neurology.

[2] Andersen OM, Reiche J, Schmidt V, Gotthardt M, Spoelgen R, Behlke J (2005). Neuronal sorting protein-related receptor sorLA/LR11 regulates processing of the amyloid precursor protein. Proc Natl Acad Sci U S A.

[3] Arvanitakis Z, Schneider JA, Wilson RS, Li Y, Arnold SE, Wang Z (2006). Diabetes is related to cerebral infarction but not to AD pathology in older persons. Neurology.

[4] Baker LD, Cross DJ, Minoshima S, Belongia D, Watson GS, Craft S (2011). Insulin resistance and Alzheimer-like reductions in regional cerebral glucose metabolism for cognitively normal adults with prediabetes or early type 2 diabetes. Arch Neurol.

[5] Barnes DE, Yaffe K (2011). The projected effect of risk factor reduction on Alzheimer’s disease prevalence. Lancet Neurol.

[6] Beckett PR (2000). Spectrophotometric assay for measuring branched-chain amino acids. Methods Enzymol.

[7] Beeri MS, Silverman JM, Davis KL, Marin D, Grossman HZ, Schmeidler J (2005). Type 2 diabetes is negatively associated with Alzheimer’s disease neuropathology. J Gerontol A Biol Sci Med Sci.

[8] Cao D, Lu H, Lewis TL, Li L (2007). Intake of sucrose-sweetened water induces insulin resistance and exacerbates memory deficits and amyloidosis in a transgenic mouse model of Alzheimer disease. J Biol Chem.

[9] Clarke JR, Lyra ESNM, Figueiredo CP, Frozza RL, Ledo JH, Beckman D, Katashima CK, Razolli D, Carvalho BM, Frazao Ret al (2015) Alzheimer-associated Abeta oligomers impact the central nervous system to induce peripheral metabolic deregulation. EMBO molecular medicine 7: 190-210 Doi 10.15252/emmm.201404183 10.15252/emmm.201404183 PMC4328648 25617315

[10] Clee SM, Yandell BS, Schueler KM, Rabaglia ME, Richards OC, Raines SM (2006). Positional cloning of Sorcs1, a type 2 diabetes quantitative trait locus. Nat Genet.

[11] Corder EH, Saunders AM, Strittmatter WJ, Schmechel DE, Gaskell PC, Small GW (1993). Gene dose of apolipoprotein E type 4 allele and the risk of Alzheimer’s disease in late onset families. Science.

[12] Dodson SE, Gearing M, Lippa CF, Montine TJ, Levey AI, Lah JJ (2006). LR11/SorLA expression is reduced in sporadic Alzheimer disease but not in familial Alzheimer disease. J Neuropathol Exp Neurol.

[13] Goodarzi MO, Lehman DM, Taylor KD, Guo X, Cui J, Quinones MJ (2007). SORCS1: a novel human type 2 diabetes susceptibility gene suggested by the mouse. Diabetes.

[14] Granhall C, Park HB, Fakhrai-Rad H, Luthman H (2006). High-resolution quantitative trait locus analysis reveals multiple diabetes susceptibility loci mapped to intervals < 800 kb in the species-conserved Niddm1i of the GK rat. Genetics.

[15] Haan MN (2006). Therapy Insight: type 2 diabetes mellitus and the risk of late-onset Alzheimer’s disease. Nat Clin Pract Neurol.

[16] Heitner J, Dickson D (1997). Diabetics do not have increased Alzheimer-type pathology compared with age-matched control subjects. A retrospective postmortem immunocytochemical and histofluorescent study. Neurology.

[17] Hiltunen M, Khandelwal VK, Yaluri N, Tiilikainen T, Tusa M, Koivisto H (2012). Contribution of genetic and dietary insulin resistance to Alzheimer phenotype in APP/PS1 transgenic mice. J Cell Mol Med.

[18] Ho L, Qin W, Pompl PN, Xiang Z, Wang J, Zhao Z (2004). Diet-induced insulin resistance promotes amyloidosis in a transgenic mouse model of Alzheimer’s disease. FASEB J.

[19] Janson J, Laedtke T, Parisi JE, O’Brien P, Petersen RC, Butler PC (2004). Increased risk of type 2 diabetes in Alzheimer disease. Diabetes.

[20] Jimenez-Palomares M, Ramos-Rodriguez JJ, Lopez-Acosta JF, Pacheco-Herrero M, Lechuga-Sancho AM, Perdomo G (2012). Increased Abeta production prompts the onset of glucose intolerance and insulin resistance. Am J Physiol Endocrinol Metab.

[21] Kalmijn S, Launer LJ, Ott A, Witteman JC, Hofman A, Breteler MM (1997). Dietary fat intake and the risk of incident dementia in the Rotterdam Study. Ann Neurol.

[22] Kang DE, Saitoh T, Chen X, Xia Y, Masliah E, Hansen LA (1997). Genetic association of the low-density lipoprotein receptor-related protein gene (LRP), an apolipoprotein E receptor, with late-onset Alzheimer’s disease. Neurology.

[23] Kebede MA, Oler AT, Gregg T, Balloon AJ, Johnson A, Mitok K (2014). SORCS1 is necessary for normal insulin secretory granule biogenesis in metabolically stressed beta cells. J Clin Invest.

[24] Killick R, Scales G, Leroy K, Causevic M, Hooper C, Irvine EE (2009). Deletion of Irs2 reduces amyloid deposition and rescues behavioural deficits in APP transgenic mice. Biochem Biophys Res Commun.

[25] Knight EM, Martins IV, Gumusgoz S, Allan SM, Lawrence CB (2014). High-fat diet-induced memory impairment in triple-transgenic Alzheimer’s disease (3xTgAD) mice is independent of changes in amyloid and tau pathology. Neurobiol Aging.

[26] Knight EM, Williams HN, Stevens AC, Kim SH, Kottwitz JC, Morant AD (2015). Evidence that small molecule enhancement of beta-hexosaminidase activity corrects the behavioral phenotype in Dutch APP(E693Q) mice through reduction of ganglioside-bound Abeta. Mol Psychiatry.

[27] Kuusisto J, Koivisto K, Mykkanen L, Helkala EL, Vanhanen M, Hanninen T (1997). Association between features of the insulin resistance syndrome and Alzheimer’s disease independently of apolipoprotein E4 phenotype: cross sectional population based study. BMJ.

[28] Lambert MP, Velasco PT, Chang L, Viola KL, Fernandez S, Lacor PN (2007). Monoclonal antibodies that target pathological assemblies of Abeta. J Neurochem.

[29] Lane RF, Raines SM, Steele JW, Ehrlich ME, Lah JA, Small SA (2010). Diabetes-associated SorCS1 regulates Alzheimer’s amyloid-beta metabolism: evidence for involvement of SorL1 and the retromer complex. J Neurosci.

[30] Leibson CL, Rocca WA, Hanson VA, Cha R, Kokmen E, O’Brien PC (1997). Risk of dementia among persons with diabetes mellitus: a population-based cohort study. Am J Epidemiol.

[31] Lendon CL, Talbot CJ, Craddock NJ, Han SW, Wragg M, Morris JC (1997). Genetic association studies between dementia of the Alzheimer’s type and three receptors for apolipoprotein E in a Caucasian population. Neurosci Lett.

[32] Li W, Prakash R, Kelly-Cobbs AI, Ogbi S, Kozak A, El-Remessy AB (2010). Adaptive cerebral neovascularization in a model of type 2 diabetes: relevance to focal cerebral ischemia. Diabetes.

[33] Liang X, Slifer M, Martin ER, Schnetz-Boutaud N, Bartlett J, Anderson B (2009). Genomic convergence to identify candidate genes for Alzheimer disease on chromosome 10. Hum Mutat.

[34] Matsuzaki T, Sasaki K, Tanizaki Y, Hata J, Fujimi K, Matsui Y (2010). Insulin resistance is associated with the pathology of Alzheimer disease: the Hisayama study. Neurology.

[35] Mody N, Agouni A, McIlroy GD, Platt B, Delibegovic M (2011). Susceptibility to diet-induced obesity and glucose intolerance in the APP (SWE)/PSEN1 (A246E) mouse model of Alzheimer’s disease is associated with increased brain levels of protein tyrosine phosphatase 1B (PTP1B) and retinol-binding protein 4 (RBP4), and basal phosphorylation of S6 ribosomal protein. Diabetologia.

[36] Muhammad A, Flores I, Zhang H, Yu R, Staniszewski A, Planel E (2008). Retromer deficiency observed in Alzheimer’s disease causes hippocampal dysfunction, neurodegeneration, and Abeta accumulation. Proc Natl Acad Sci U S A.

[37] Murakami K, Yokoyama S, Murata N, Ozawa Y, Irie K, Shirasawa T (2011). Insulin receptor mutation results in insulin resistance and hyperinsulinemia but does not exacerbate Alzheimer’s-like phenotypes in mice. Biochem Biophys Res Commun.

[38] Niedowicz DM, Reeves VL, Platt TL, Kohler K, Beckett TL, Powell DK (2014). Obesity and diabetes cause cognitive dysfunction in the absence of accelerated beta-amyloid deposition in a novel murine model of mixed or vascular dementia. Acta Neuropathol Commun.

[39] Nielsen MS, Gustafsen C, Madsen P, Nyengaard JR, Hermey G, Bakke O (2007). Sorting by the cytoplasmic domain of the amyloid precursor protein binding receptor SorLA. Mol Cell Biol.

[40] Nishimura N, Hachisuga T, Saito T, Kawarabayashi T (2001). Subsequent endometrial carcinoma with adjuvant tamoxifen treatment in Japanese breast cancer patients. Int J Gynecol Cancer.

[41] Offe K, Dodson SE, Shoemaker JT, Fritz JJ, Gearing M, Levey AI (2006). The lipoprotein receptor LR11 regulates amyloid beta production and amyloid precursor protein traffic in endosomal compartments. J Neurosci.

[42] Ott A, Stolk RP, van Harskamp F, Pols HA, Hofman A, Breteler MM (1999). Diabetes mellitus and the risk of dementia: The Rotterdam Study. Neurology.

[43] Peila R, Rodriguez BL, Launer LJ, Honolulu-Asia Aging S (2002). Type 2 diabetes, APOE gene, and the risk for dementia and related pathologies: The Honolulu-Asia Aging Study. Diabetes.

[44] Pettersson US, Walden TB, Carlsson PO, Jansson L, Phillipson M (2012). Female mice are protected against high-fat diet induced metabolic syndrome and increase the regulatory T cell population in adipose tissue. PLoS One.

[45] Pezzini A, Grassi M, Paciaroni M, Zini A, Silvestrelli G, Iacoviello L (2013). Obesity and the risk of intracerebral hemorrhage: the multicenter study on cerebral hemorrhage in Italy. Stroke.

[46] Plaschke K, Kopitz J, Siegelin M, Schliebs R, Salkovic-Petrisic M, Riederer P (2010). Insulin-resistant brain state after intracerebroventricular streptozotocin injection exacerbates Alzheimer-like changes in Tg2576 AbetaPP-overexpressing mice. J Alzheimers Dis.

[47] Prakash R, Johnson M, Fagan SC, Ergul A (2013). Cerebral neovascularization and remodeling patterns in two different models of type 2 diabetes. PLoS One.

[48] Prakash R, Somanath PR, El-Remessy AB, Kelly-Cobbs A, Stern JE, Dore-Duffy P (2012). Enhanced cerebral but not peripheral angiogenesis in the Goto-Kakizaki model of type 2 diabetes involves VEGF and peroxynitrite signaling. Diabetes.

[49] Printy BP, Verma N, Cowperthwaite MC, Markey MK, Alzheimer’s Disease Neuroimaging I (2014). Effects of genetic variation on the dynamics of neurodegeneration in Alzheimer’s disease. Conf Proc IEEE Eng Med Biol Soc.

[50] Ramos-Rodriguez JJ, Infante-Garcia C, Galindo-Gonzalez L, Garcia-Molina Y, Lechuga-Sancho A, Garcia-Alloza M (2015) Increased Spontaneous Central Bleeding and Cognition Impairment in APP/PS1 Mice with Poorly Controlled Diabetes Mellitus. Molecular neurobiology: Doi 10.1007/s12035-015-9311-2 10.1007/s12035-015-9311-2 PMC4823354 26156287

[51] Ramos-Rodriguez JJ, Jimenez-Palomares M, Murillo-Carretero MI, Infante-Garcia C, Berrocoso E, Hernandez-Pacho F (2015). Central vascular disease and exacerbated pathology in a mixed model of type 2 diabetes and Alzheimer’s disease. Psychoneuroendocrinology.

[52] Ramos-Rodriguez JJ, Ortiz-Barajas O, Gamero-Carrasco C, de la Rosa PR, Infante-Garcia C, Zopeque-Garcia N (2014). Prediabetes-induced vascular alterations exacerbate central pathology in APPswe/PS1dE9 mice. Psychoneuroendocrinology.

[53] Reitz C, Tokuhiro S, Clark LN, Conrad C, Vonsattel JP, Hazrati LN (2011). SORCS1 alters amyloid precursor protein processing and variants may increase Alzheimer’s disease risk. Ann Neurol.

[54] Ristow M (2004). Neurodegenerative disorders associated with diabetes mellitus. J Mol Med (Berl).

[55] Rogaeva E, Meng Y, Lee JH, Gu Y, Kawarai T, Zou F (2007). The neuronal sortilin-related receptor SORL1 is genetically associated with Alzheimer disease. Nat Genet.

[56] Ruiz HH CT, Lindtner C, Hsieh W, Ehrlich M, Gandy S, Buettner C. (2016) Increased susceptibility to metabolic dysregulation in a mouse model of Alzheimer’s disease is associated with impaired hypothalamic insulin signaling and elevated BCAA levels. Alzheimer’s and Dementia, in press 10.1016/j.jalz.2016.01.008 PMC5358328 26928090

[57] Sadahiro M, Erickson C, Lin WJ, Shin AC, Razzoli M, Jiang C (2015). Role of VGF-derived carboxy-terminal peptides in energy balance and reproduction: analysis of “humanized” knockin mice expressing full-length or truncated VGF. Endocrinology.

[58] Sager KL, Wuu J, Leurgans SE, Rees HD, Gearing M, Mufson EJ (2007). Neuronal LR11/sorLA expression is reduced in mild cognitive impairment. Ann Neurol.

[59] Saunders AM, Strittmatter WJ, Schmechel D, George-Hyslop PH, Pericak-Vance MA, Joo SH (1993). Association of apolipoprotein E allele epsilon 4 with late-onset familial and sporadic Alzheimer’s disease. Neurology.

[60] Scherzer CR, Offe K, Gearing M, Rees HD, Fang G, Heilman CJ (2004). Loss of apolipoprotein E receptor LR11 in Alzheimer disease. Arch Neurol.

[61] Schmidt V, Sporbert A, Rohe M, Reimer T, Rehm A, Andersen OM (2007). SorLA/LR11 regulates processing of amyloid precursor protein via interaction with adaptors GGA and PACS-1. J Biol Chem.

[62] Schrijvers EM, Witteman JC, Sijbrands EJ, Hofman A, Koudstaal PJ, Breteler MM (2010). Insulin metabolism and the risk of Alzheimer disease: the Rotterdam Study. Neurology.

[63] Sims-Robinson C, Kim B, Rosko A, Feldman EL (2010). How does diabetes accelerate Alzheimer disease pathology?. Nat Rev Neurol.

[64] Small SA, Kent K, Pierce A, Leung C, Kang MS, Okada H (2005). Model-guided microarray implicates the retromer complex in Alzheimer’s disease. Ann Neurol.

[65] Strachan MW, Reynolds RM, Frier BM, Mitchell RJ, Price JF (2008). The relationship between type 2 diabetes and dementia. Br Med Bull.

[66] Takeda S, Sato N, Uchio-Yamada K, Sawada K, Kunieda T, Takeuchi D (2010). Diabetes-accelerated memory dysfunction via cerebrovascular inflammation and Abeta deposition in an Alzheimer mouse model with diabetes. Proc Natl Acad Sci U S A.

[67] Tomic JL, Pensalfini A, Head E, Glabe CG (2009). Soluble fibrillar oligomer levels are elevated in Alzheimer’s disease brain and correlate with cognitive dysfunction. Neurobiol Dis.

[68] Willette AA, Johnson SC, Birdsill AC, Sager MA, Christian B, Baker LD (2015). Insulin resistance predicts brain amyloid deposition in late middle-aged adults. Alzheimer’s Dement.

[69] Xu WL, Qiu CX, Wahlin A, Winblad B, Fratiglioni L (2004). Diabetes mellitus and risk of dementia in the Kungsholmen project: a 6-year follow-up study. Neurology.

[70] Zhang Y, Zhou B, Deng B, Zhang F, Wu J, Wang Y (2013). Amyloid-beta induces hepatic insulin resistance in vivo via JAK2. Diabetes.

