# Supplementary material for: Unexpected partial correction of metabolic and behavioral phenotypes of Alzheimer’s APP/PSEN1 mice by gene targeting of diabetes/Alzheimer’s-related Sorcs1
Source: Acta Neuropathol Commun. 2016 Feb 25;4:16. doi: 10.1186/s40478-016-0282-y (PMC4766719; doi:10.1186/s40478-016-0282-y)

Additional file 1: Figure S1. Young *Sorcs1* -/-, *APP/PSEN1* mice and *Sorcs1* -/- x *APP/PSEN1* mice display normal glucose homeostasis. Glucose tolerance testing **(a,c)**, fasting plasma insulin **(b,d)**, body weight **(e,g)**, body length **(f,h)**, fasting plasma glycerol **(i,k)**, fasting plasma triglycerides **(j,l)**, lean mass **(m,o)** and fat mass **(n,p)** in cohorts of male and female WT (n=5-8/group), *Sorcs1* -/- (n=10/group), *APP/PSEN1* (n=4-8/group) and *Sorcs1* -/- x *APP/PSEN1* (n=4-6/group). Cohorts were maintained on standard rodent chow from weaning and assessed at 4-5 months of age. *$P<0.05$, **$P<0.01$ and ***$P<0.001$; One-way ANOVA with Bonferroni posthoc analyses. Data expressed as mean ± s.e.m.

Additional file 1: Figure S1

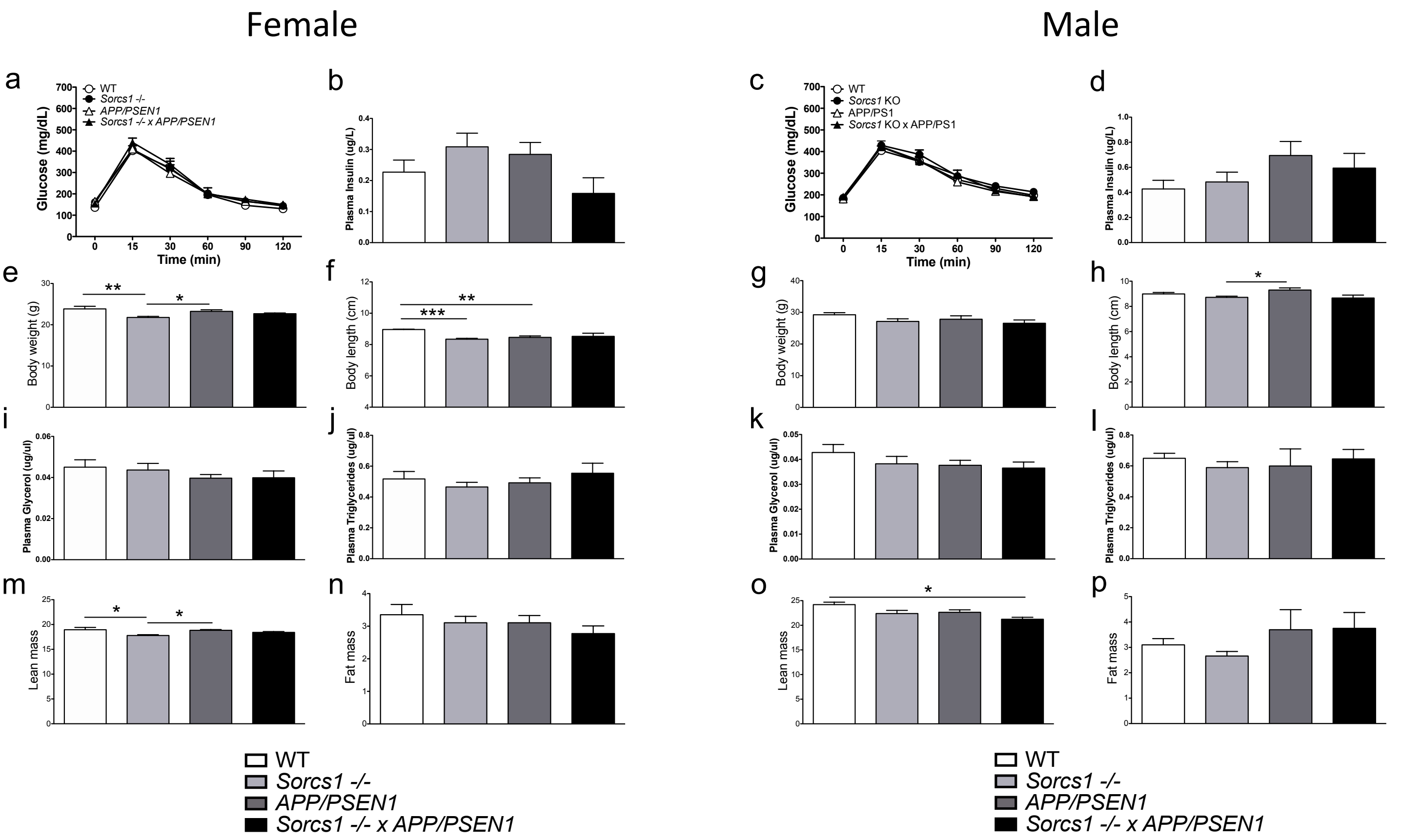

Supplement: Additional file 1: Figure S1. — Young Sorcs1 -/-, APP/PSEN1 mice and Sorcs1 -/- x APP/PSEN1 mice display normal glucose homeostasis. Glucose tolerance testing (a,c), fasting plasma insulin (b,d), body weight (e,g), body length (f,h), fasting plasma glycerol (i,k), fasting plasma triglycerides (j,l), lean mass (m,o) and fat mass (n,p) in cohorts of male and female WT (n = 5- 8/group), Sorcs1 -/- (n = 10/group), APP/PSEN1 (n = 4-8/group) and Sorcs1 -/- x APP/PSEN1 (n = 4- 6/group). Cohorts were maintained on standard rodent chow from weaning and assessed at 4-5 months of age. *P < 0.05, **P < 0.01 and ***P < 0.001; One-way ANOVA with Bonferroni posthoc analyses. Data expressed as mean ± s.e.m. (PDF 350 kb) [file 40478_2016_282_MOESM1_ESM.pdf]
